# Use of the *kojA* promoter, involved in kojic acid biosynthesis, for polyketide production in *Aspergillus oryzae*: implications for long-term production

**DOI:** 10.1186/s12896-019-0567-x

**Published:** 2019-10-26

**Authors:** Koichi Tamano, Mahoko Kuninaga, Naoshi Kojima, Myco Umemura, Masayuki Machida, Hideaki Koike

**Affiliations:** 1 Bioproduction Research Institute, National Institute of Advanced Industrial Science and Technology (AIST), 2-17-2-1 Tsukisamu-Higashi, Toyohira-ku, Sapporo, Hokkaido 062-8517 Japan; 2 AIST-Waseda University Computational Bio Big-Data Open Innovation Laboratory (CBBD-OIL), National Institute of Advanced Industrial Science and Technology (AIST), 5-20, Building 63, Nishi-waseda campus, Waseda University, 3-4-1 Okubo, Shinjuku-ku, Tokyo, 169-8555 Japan; 3 Bioproduction Research Institute, National Institute of Advanced Industrial Science and Technology (AIST), Central 6, 1-1-1 Higashi, Tsukuba, Ibaraki, 305-8566 Japan; 4 Biomedical Research Institute, National Institute of Advanced Industrial Science and Technology (AIST), Central 6, 1-1-1 Higashi, Tsukuba, Ibaraki, 305-8566 Japan

**Keywords:** *Aspergillus oryzae*, Kojic acid, Secondary metabolism, Transcriptional regulation, Polyketide

## Abstract

**Background:**

*Aspergillus oryzae*, a useful industrial filamentous fungus, produces limited varieties of secondary metabolites, such as kojic acid. Thus, for the production of valuable secondary metabolites by genetic engineering, the species is considered a clean host, enabling easy purification from cultured cells. *A. oryzae* has been evaluated for secondary metabolite production utilizing strong constitutive promoters of genes responsible for primary metabolism. However, secondary metabolites are typically produced by residual nutrition after microbial cells grow to the stationary phase and primary metabolism slows. We focused on a promoter of the secondary metabolism gene *kojA*, a component of the kojic acid biosynthetic gene cluster, for the production of other secondary metabolites by *A. oryzae*.

**Results:**

A *kojA* disruptant that does not produce kojic acid was utilized as a host strain for production. Using this host strain, a mutant that expressed a polyketide synthase gene involved in polyketide secondary metabolite production under the *kojA* gene promoter was constructed. Then, polyketide production and polyketide synthase gene expression were observed every 24 h in liquid culture. From days 0 to 10 of culture, the polyketide was continuously produced, and the synthase gene expression was maintained. Therefore, the *kojA* promoter was activated, and it enabled the continuous production of polyketide for 10 days.

**Conclusions:**

The combined use of the *kojA* gene promoter and a *kojA* disruptant proved useful for the continuous production of a polyketide secondary metabolite in *A. oryzae.* These findings suggest that this combination can be applied to other secondary metabolites for long-term production.

## Background

*Aspergillus oryzae* is a useful industrial filamentous fungus; it has been utilized for the production of Japanese alcohol (*sake*), soy sauce (*shoyu*), soybean paste (*miso*), and other products since ancient periods in Japan. It has a strong ability to produce hydrolases, such as amylase, protease, and lipase. It is also beneficial with respect to safety owing to its lack of mycotoxin production and formally approved as a safe microorganism with the GRAS (Generally Recognized as Safe) status by FDA (Food and Drug Administration of USA). The whole genome sequence of the wild-type *A. oryzae* strain RIB40 was reported in 2005 [1], providing a basis for targeted gene disruption or overexpression [2, 3]. In addition to enzymes, industrially valuable metabolite production has been attempted using *A. oryzae* by metabolic engineering via genetic modification technology [4–6].

*A. oryzae* produces a limited array of secondary metabolites, such as kojic acid [7, 8], and does not produce mycotoxins, such as aflatoxin and cyclopiazonic acid [9, 10]. In fact, heterologous production of secondary metabolites of polyketides, terpenes, and non-ribosomal peptides has ever been attempted in *A. oryzae*, and from the data presented here, it is indicated that none or only one peak of compounds with similar physical properties exist in the wild-type strain [11–14]. Thus, for the heterologous production of valuable secondary metabolites, we proposed *A. oryzae* to be adequate with respect to the ease of purification from cultured cells. In other words, it should be expected to be a clean host with limited endogenous secondary metabolites. Furthermore, knockout mutants of kojic acid biosynthesis genes are considered more effective hosts for secondary metabolite production than the wild-type strain because they do not produce any kojic acid.

Fungal secondary metabolites are required in the cells or spores for protection against ultraviolet (UV) and other microorganisms like bacteria, or for development [15]. Some of them possess medicinal properties and are actually used as pharmaceuticals such as penicillin, lovastatin, and cyclosporin [15, 16]. Most secondary metabolites are categorized into three groups: polyketides, small peptides (e.g. non-ribosomal peptides), and terpenes [15, 17]. Polyketides and terpenes are synthesized from acetyl-CoA and malonyl-CoA, and thus are characteristically composed of only carbon, oxygen, and hydrogen, but not nitrogen. Non-ribosomal peptides are synthesized from amino acids and thus contain nitrogen as well. Kojic acid shares the components of polyketides and terpenes; however, it is considered not to be categorized as either of them and considered to be synthesized through a different metabolic pathway [18].

**Fig. 1:**
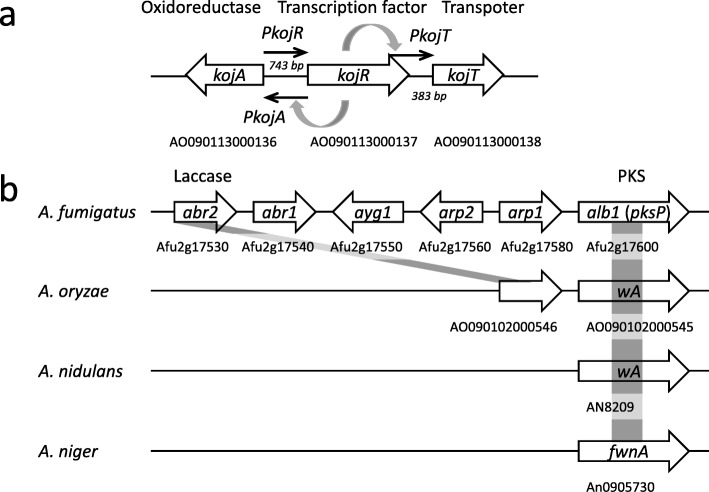
Maps of the kojic acid biosynthesis cluster and the polyketide synthase (PKS) gene with the surrounding genes associated with spore pigment biosynthesis in *Aspergillus* species. Each gene is shown by box arrow. The estimated function, systematic gene name, and gene ID are denoted above, in, and below the box arrow, respectively. (**a**) The kojic acid biosynthesis cluster composed of three genes. Each promoter is shown by arrow. (**b**) Four species that are *A. fumigatus*, *A. oryzae*, *A. nidulans*, and *A. niger* are used for the mapping. The PKS genes are conserved among all four species, whereas genes of the other orthologs composing the spore pigment biosynthesis cluster in *A. fumigatus* are not almost located around the PKS gene in the other species. Only the laccase gene in *A. oryzae* is exceptionally located adjacent to the PKS gene

In fact, for the first time, we previously identified genes responsible for kojic acid biosynthesis in *A. oryzae* [19]. They comprise *kojA* (AO090113000136), *kojR* (AO090113000137), and *kojT* (AO090113000138) and form a gene cluster on the chromosome (Fig. 1a) [19]. To date, it is revealed that the *kojR*-translated product, KojR, acts as a transcription factor and can activate promoters of other cluster genes, namely *kojA* and *kojT* [20]. The *kojA*-translated product, KojA, is predicted to be an oxidoreductase and directly contributes to kojic acid biosynthesis using some sort of metabolite as a substrate. However, it does not have a motif sequence of polyketide synthase (PKS) and P450 that usually exist in polyketide and terpene biosynthesis gene clusters, respectively. The *kojT*-translated product, KojT, is predicted to function as a transporter located on the cell membrane and transport kojic acid to the extracellular environment. These findings also indicate that kojic acid biosynthesis will be independent of polyketide and terpene biosyntheses.

We also analyzed the culture conditions under which this biosynthesis is activated. Kojic acid was continuously synthesized from culture day 3 to day 10 in kojic acid production liquid medium using residual glucose as a substrate after the nitrogen source was completely catabolized [19]. This implies that kojic acid biosynthesis is suppressed in the presence of a nitrogen source. In fact, the *kojA* gene expression was found to be repressed until the nitrogen source was used up [19]. Kojic acid is not essential for cell growth and thus is considered a secondary metabolite. Therefore, the promoters in the kojic acid biosynthesis gene cluster should exhibit lower activity levels than those of primary metabolism genes. However, because kojic acid is continuously produced, promoters of the genes involved in its biosynthesis are expected to be activated for a long period during production. In the stationary growth phase of *A. oryzae*, the primary metabolism diminishes, and kojic acid accumulates in the cells owing to its continuous production using the residual carbon source, glucose [19]. In such a situation, selective production of a metabolite seems to come into play, which will contribute to the easy and efficient recovery from cells. Therefore, in this study, we focused on the long-term production characteristics of kojic acid, and a promoter of *kojA* was evaluated for the long-term production of other secondary metabolites in *A. oryzae*. The polyketide synthase gene *wA* of *A. oryzae* was chosen for the evaluation, because polyketide has the same composition as kojic acid and could be easily quantified by a specific ultraviolet–visible (UV-Vis) absorbance.

## Results

### Production of a polyketide YWA1 synthesized by *A. oryzae wA* under the *kojA* promoter in the *kojA* disruptant

**Fig. 2:**
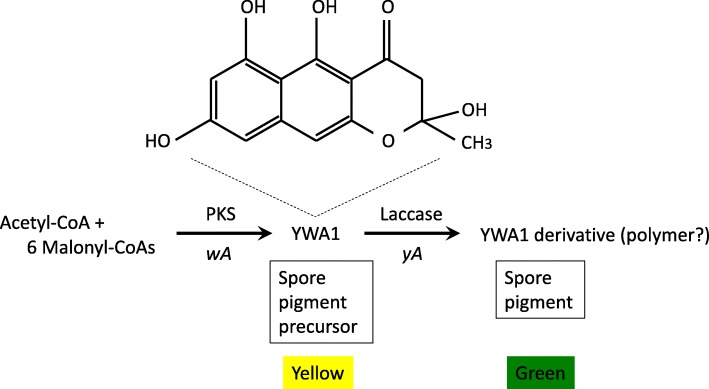
Illustration of biosynthetic pathway of spore pigment in *A. nidulans*. The first step is performed by PKS encoded by *A. nidulans wA*. The polyketide named YWA1 is synthesized and the structure is drawn above. The second step is performed by a laccase encoded by *yA*. The similar pathway seems to be there in *A. oryzae*

To begin with, we validated our assumption that the expression of primary metabolism genes attenuates toward the end of culture. The *enoA* gene encoding the enolase enzyme in glycolysis is considered constitutive gene and has ever been used for heterologous gene expression [21]. It was selected for the validation. The expression was certainly decreased as the culture of RIB40 proceeded in the kojic acid production medium (Additional file 1: Figure S1). Thus, our assumption seemed adequate, and as mentioned above, we then chose a polyketide secondary metabolite for evaluating the applicability of the *kojA* promoter to the production. It is because the *kojA* promoter activation is dependent on a nitrogen-limited culture environment. Polyketide does not contain nitrogen in the molecule, and thus it was considered appropriate for evaluating the expression system by the *kojA* promoter. In the *A. oryzae* genome, orthologues of genes involved in the biosynthesis of the spore pigment were predicted (Fig. 1b). A PKS encoded by *wA* (AO090102000545), an ortholog of *alb1* (Afu2g17600) in *Aspergillus fumigatus* Af293, *fwnA* (An09g05730) in *Aspergillus niger* CBS 513.88, and *wA* (AN8209) in *Aspergillus nidulans* A4, is likely involved in the initial step of biosynthesis of spore pigments using malonyl-CoAs as substrates. According to a previous study based on *A. nidulans* (Fig. 2), the product of *A. nidulans wA* is a spore pigment precursor; however, it has a yellow color and is named YWA1 [22]. The product of *A. oryzae wA* was expected to be similar to that of *A. nidulans wA* and expected to be measured by absorbance easily in the experimental technique. Thus, by replacing the promoter of *wA* with that of *kojA*, production of the polyketide derived from *A. oryzae wA* under the regulation of the *kojA* promoter was attempted in *A. oryzae*.

**Fig. 3:**
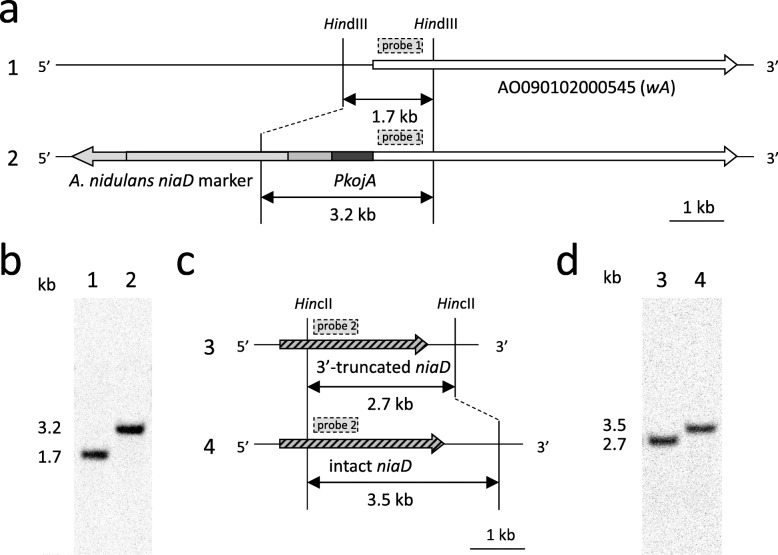
Construction and confirmation of the *ΔkojA_PkojA::wA* and *ΔkojA* strains. (**a**) Change in the *wA* promoter region during construction of the *ΔkojA_PkojA* strain. (**b**) Construction of the *ΔkojA_PkojA* strain was confirmed by Southern hybridization. The site where the probe 1 hybridizes is shown. 1: *ΔkojA*, 2: *ΔkojA_PkojA::wA*. (**c**) Complementation of the *niaD* during construction of the *ΔkojA* strain. (**d**) Construction of the *ΔkojA* strain was confirmed by Southern hybridization. The site where the probe 2 hybridizes is shown. 3: *ΔkojA_niaD-*, 4: *ΔkojA*

**Fig. 4:**
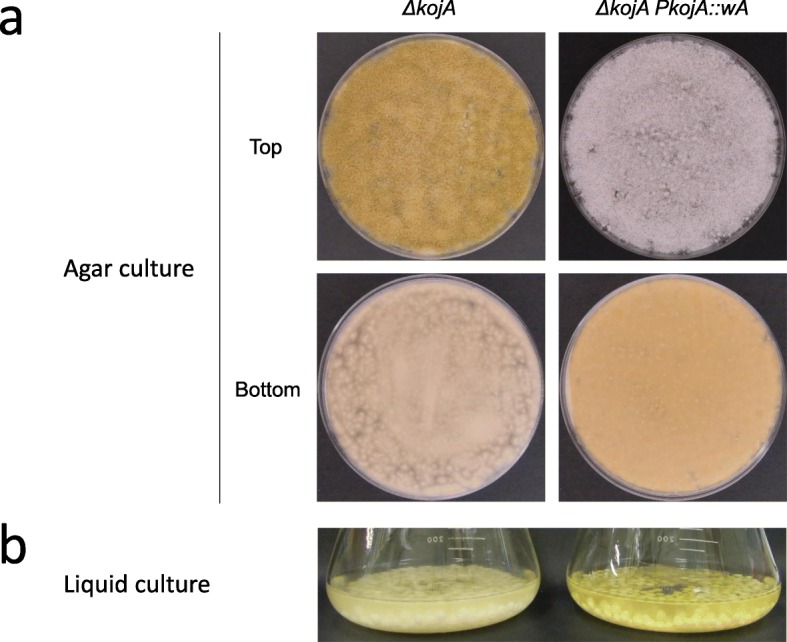
Images of the *ΔkojA* (left) and the *ΔkojA_PkojA::wA* (right) strains. (**a**) Image obtained after incubation at 30 °C for 7 days of the CD agar plates where 5 × 10^5^ spores were spread-inoculated. Images of plates taken from the top (upper row) or the bottom (lower row) are shown. (**b**) Image of the liquid culture after cultivation at 30 °C at 200 rpm for 5 days

**Fig. 5:**
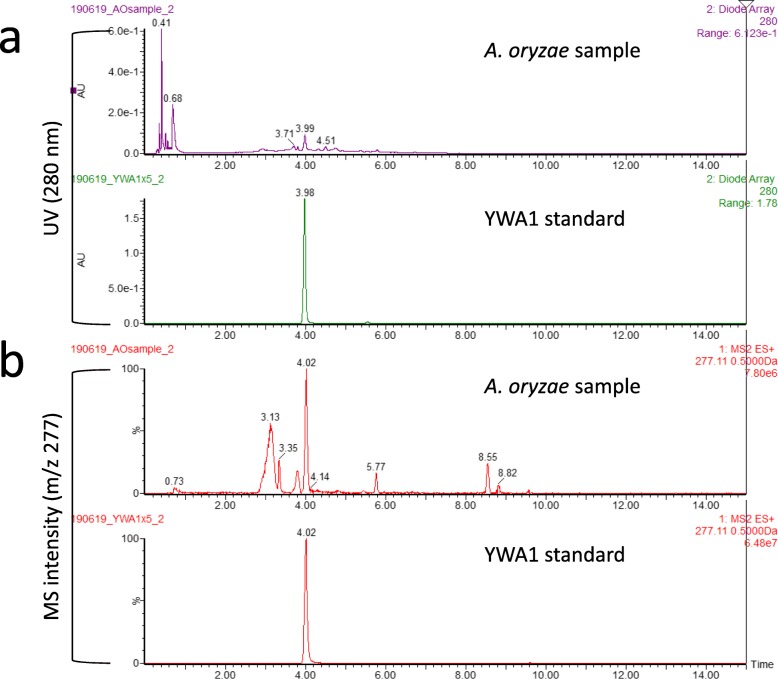
Analysis of the yellow-colored compound produced by the *ΔkojA_PkojA::wA* strain by UPLC-MS. The chromatograms of the yellow-colored compound denoted as *A. oryzae* sample and the YWA1 standard by UPLC (**a**) and MS (**b**) are shown. The UPLC chromatograms are taken at 280 nm, while the MS chromatograms are taken at m/z: 277. X-axes; retention time (min): Y-axes; absorbance at 280 nm (**a**) and signal intensity (**b**)

A *kojA* disruptant constructed in our previous study and named *ΔkojA_niaD-* [19] was used as a parental host strain for constructing the mutant strain expressing *wA* under the control of the *kojA* promoter. The *ΔkojA_niaD-* strain does not produce kojic acid and should produce only small amounts of other secondary metabolites. The *ΔkojA_niaD-* strain is also an auxotroph of nitrate due to a mutation in a nitrate reductase gene (*niaD*, AO090012001035); thus, *niaD* was used as a selectable marker. A DNA cassette for the expression of *A. oryzae wA* under the *kojA* promoter (Additional file 1: Figure S2) was prepared by fusion PCR as described in the Methods section. It was introduced to the *ΔkojA_niaD-* strain by transformation, and the transformant colonies were subjected to single spore isolation and verified by both PCR and Southern hybridization. Homologous recombination was confirmed at only the targeted *wA* locus by Southern hybridization (Fig. 3a-b). The mutant with promoter replacement was named *ΔkojA_PkojA::wA*. Similarly, the entire intact *niaD* gene was prepared by PCR using RIB40 chromosomal DNA as a template (Additional file 1: Figure S3), and was introduced to the *ΔkojA_niaD-* strain to construct a control mutant strain. The *niaD*-complemented *kojA* disruptant was confirmed by Southern hybridization (Fig. 3c-d) and was named *ΔkojA*. Both mutant strains were cultured on kojic acid production agar. The *ΔkojA* strain showed yellow-green spores, similar to the spores of the wild-type strain, whereas the *ΔkojA_PkojA::wA* strain showed white spores (Fig. 4a, upper row). In contrast, the agar became yellowish only for the *ΔkojA_PkojA::wA* strain (Fig. 4A, lower row). When cultured in kojic acid production liquid medium, the culture supernatant of the *ΔkojA_PkojA::wA* strain became more yellowish than the supernatant of the *ΔkojA* strain (Fig. 4b). Considering that this yellow color was derived from PKS, extraction and confirmation of the yellow-colored compound were attempted using ethyl acetate. The UV-Vis spectrum corresponded to that of the YWA1 standard (Additional file 1: Figures S4). A UPLC-MS analysis was also performed for the sample and the standard. The retention time of the major peak at 4.0 min observed at 280 nm by UPLC superimposed with that of YWA1 standard (Fig. 5a). In addition, the MS chromatogram of sample compounds whose m/z was 277 was identical to that of the YWA1 standard (Fig. 5b). Hence, taken together, the yellow-colored compound produced by the *ΔkojA_PkojA::wA* strain was attributed to the polyketide synthase encoded by *wA* and concluded to be YWA1.

### Time-course shifts in *wA* expression under the *kojA* promoter and the polyketide yield

**Fig. 6:**
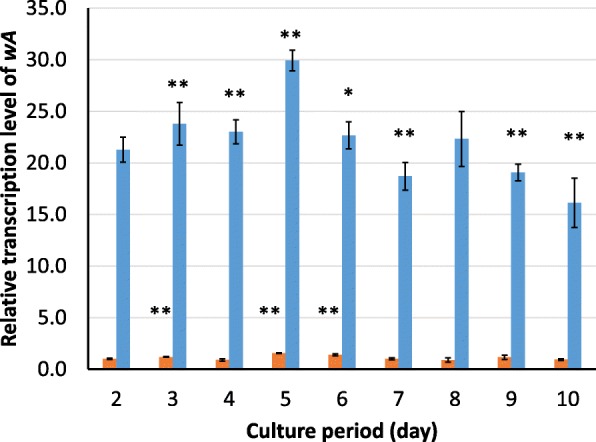
Time-course shift in *A. oryzae wA* expression. Expression levels of *wA* in the wild-type RIB40 (orange) and the *ΔkojA_PkojA::wA* (blue) strains at each time point relative to that on day 2 of RIB40 are shown. Error bars represent mean ± standard deviation (*n* = 3). The result of a significance test (Student’s t-test) between day 2 and the other date is shown above each bar. *and ** represent *p* < 0.05 and *p* < 0.01, respectively

The activation of the *kojA* promoter was evaluated in the *ΔkojA_PkojA::wA* strain. The expression level of *wA* and the YWA1 yield were measured every 24 h in a stepwise manner from the beginning of the culture period until day 10. As determined by qRT-PCR, *wA* remained active from day 2 to day 10 of the culture period (Fig. 6). The expression level exhibited a peak on day 5 and decreased after day 9, but the differences were minimal. Therefore, the *kojA* promoter was active up to day 10 of the culture period. In the control *ΔkojA* strain, the expression level of *wA* showed a similar trend. As a result, *wA* expression of the *ΔkojA* strain was approximately 0.05 fold or less of that of *ΔkojA_PkojA::wA* strain in each culture period. This suggests that *wA* was overexpressed by the *kojA* promoter in the *ΔkojA_PkojA::wA* strain.

**Fig. 7:**
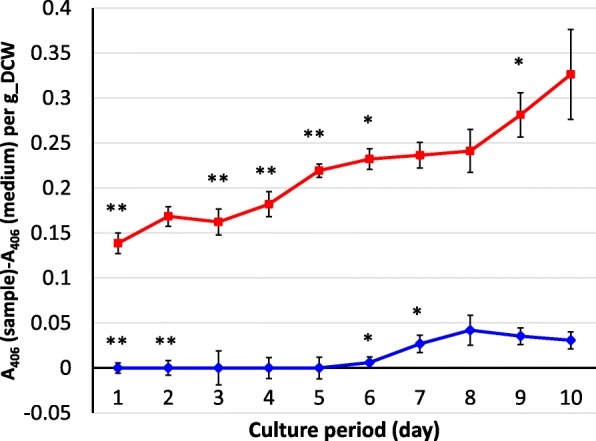
Time-course shift in the *A. oryzae wA* product YWA1 secreted by the *ΔkojA_PkojA::wA* strain. The product yield was measured by specific absorbance at 406 nm of the culture supernatant. Absorbance of the culture supernatant at each culture period was subtracted by that of culture medium without inoculation, followed by division by dry cell weight of hyphae. The calculated value was monitored for both the *ΔkojA_PkojA::wA* strain (red) and the control *ΔkojA* strain (blue). Error bars represent mean ± standard deviation (n = 3). The result of a significance test (Student’s t-test) between the day 0 and the other date is shown above each bar. * and ** represent p < 0.05 and p < 0.01, respectively

Next, YWA1 was measured by estimates of absorbance, and then the YWA1 amount produced by unit gram of dried cells was evaluated. An absorbance peak at 406 nm was detected by UV-Vis spectrometry (Additional file 1: Figure S4); accordingly, absorbance at 406 nm in the culture supernatant was measured every 24 h from the beginning of the culture period until day 10. Absorbance of the culture medium before inoculation was then subtracted from that on each culture period to remove background, and the subtracted absorbance was subsequently divided by the weight of dried cells to evaluate the amount of YWA1 produced by the same number of cells. It was observed that YWA1 was continuously produced from day 1 to day 10; production was maintained during long-term liquid culture (Fig. 7). The YWA1 amount per gram of dried cells in the *ΔkojA_PkojA::wA* strain was 10.6-fold more than that in the control strain. The results corresponded to the enhanced *wA* expression level as shown in Fig. 6, indicating that the *kojA* promoter is useful for high and long-term production. As in the case of *enoA* (Additional file 1: Figure S1), expression of constitutive genes is generally considered to attenuate toward the stationary growth phase. Though we do not directly compare the case of *wA* expression under between *kojA* promoter and *enoA* promoter, because high and long-term production was observed in case of YWA1 in this study, the *kojA* promoter seems to be useful for such a purpose.

## Discussion

In this study, we showed that the *kojA* promoter enabled the continuous production of polyketide YWA1 encoded by *wA* for 10 days in *A. oryzae*. As shown in Fig. 1b, in spite of conservation of the *wA* orthologs between four *Aspergillus* species, the other orthologs included in the melanin synthesis cluster of *A. fumigatus* do not exist or are located far apart from the *wA* ortholog on the genome in the other three *Aspergillus* species. Only the laccase gene ortholog (AO090102000546) exists adjacent to *wA* on the *A. oryzae* genome. However, another gene involved in the spore pigment biosynthesis named *yA* (AN6635) is present on the *A. nidulans* genome, which is located far away from *A. nidulans wA* on the genome. The *yA* encodes laccase as well and is reported to generate green-colored spore pigments, probably via polymerization of YWA1 through the laccase function (Fig. 2). However, the *yA* homolog of *A. oryzae* is AO090011000755 and is different from the ortholog. Thus, there seem to be two laccase candidate genes involved in the spore pigment biosynthesis in *A. oryzae*. A fact which predominantly functions in it should be researched hereafter.

Because of the white spores of the *ΔkojA_PkojA::wA* strain, the *kojA* promoter may be active only in hyphae or even in spores at lower level than the RIB40. Because the production of valuable enzymes and compounds by *A. oryzae* is performed in liquid culture, the phenomenon of whitish spores will not be a drawback when using the *kojA* promoter for production.

Because YWA1 does not contain nitrogen, the production system using the *kojA* promoter in the *kojA* disruptant could be applied to the production of other compounds lacking nitrogen, like polyketides. On the other hand, because this production system is undertaken in nitrogen source-deficient condition, it was considered difficult to be applied to the production of compounds containing nitrogen, such as non-ribosomal peptides. However, unexpectedly, YWA1 production by the *ΔkojA_PkojA::wA* strain was observed from day 1 of the culture period (Fig. 7). We expected production to begin on day 3, after the nitrogen source was catabolized. In fact, *wA* expression level under the *kojA* promoter at day 2 of the culture period was already high in the *ΔkojA_PkojA::wA* strain, while *kojA* expression level under the *kojA* promoter at the day 2 was quite low in the RIB40 strain (Additional file 1: Figure S5). The data would imply that the expression in the *ΔkojA_PkojA::wA* strain was actually independent of nitrogen starvation. The reason for this unexpected result is unclear and should be evaluated in future studies. There might be additional regulatory mechanisms apart from that utilizing the nitrogen source. For example, the *kojA* promoter was attached to the *wA* locus, where epigenetic modifications might be different from those at the original *kojA* locus. However, with respect to metabolite production, expression from the early stage of culture is advantageous and is not an issue. In addition, since the *kojA* promoter seemed active even at culture period day 1 when nitrogen source remained, although it was not exemplified in this study, the expression system with the *kojA* promoter could be applied to non-ribosomal peptide production where plenty of nitrogen source will be required in culture.

Polyketide final products are generally synthesized not only by PKS but also by several modifying enzymes. Thus, for increase in production, several genes of enzymes including PKS involved in the synthesis will need to be overexpressed. In such an attempt, multiple uses of the *kojA* promoter for expressing these genes will be one possible solution. If the *kojA* promoter was used redundantly, its transcriptional factor, KojR, would get shortage in the cell. In such a case, it will be effective to use the *kojR* gene multiply as well. Fortunately, as shown in Fig. 1a, the *kojA* promoter shares the locus with the *kojR* promoter but in the opposite direction. In other words, it is a bidirectional promoter. Thus, only extending the DNA region used for replacement with the *kojA* promoter in this study to the end of *kojR* coding sequence will enable not only the replacement but also additional *kojR* arrangement on the genome.

## Conclusions

The combination of the *kojA* gene promoter and the *kojA* disruptant was useful for continuous production of a polyketide secondary metabolite until day 10 of the culture period. The results suggested that this combination is applicable to other secondary metabolites for long-term production in *A. oryzae*.

## Methods

### Fungal strains and culture

*A. oryzae* wild-type strain RIB40 and its mutants were used. The RIB40 strain was distributed by the National Research Institute of Brewing (NRIB, Hiroshima, Japan) and approved of use for research purposes [1]. For genetic engineering, an *A. oryzae ΔkojA_niaD-* strain with the genotype *ΔligD::ptrA ΔpyrG::AnsC ΔkojA::AnpyrG* (*niaD-*), derived from NS4 [23, 24], the *niaD-* and *sC-* auxotrophic mutant of RIB40, and constructed in our previous study [19], was used. The *ΔkojA_niaD-* strain was originally removed capability of non-homologous end joining (NHEJ) due to knockout of a ligase gene *ligD* responsible for NHEJ [23]. Thus, only homologous recombination can be performed in the strain and the efficiency is quite high.

Each *A. oryzae* strain constructed by genetic engineering was maintained at 30 °C on Czapek–Dox (CD) agar medium [25], and the spores were stored in 20% (w/v) glycerol at − 80 °C until use. To test YWA1 production under the *kojA* promoter, the spores were inoculated on kojic acid production agar medium (0.25% yeast extract, 0.1% K_2_HPO_4_, 0.05% MgSO_4_-7H_2_O, 10% glucose, and 1.5% agar; pH adjusted to 6.0 before autoclaving) [19] and harvested. Then, 2.5 × 10^7^ spores were inoculated into 50 mL of the kojic acid production liquid medium and cultured at 30 °C and 200 rpm for up to 10 days.

### Construction of the *A. oryzae* mutant strains

All primers used in this study are listed in Additional file 1: Table S1. The promoter-replaced strain, *ΔkojA_PkojA::wA,* was constructed as follows. The *ΔkojA_niaD-* strain, a *kojA* disruptant lacking *niaD* constructed in our previous study [19], was transformed with the DNA fragment for *wA* expression under the *kojA* promoter. The DNA fragment was constructed by PCR and the subsequent fusion PCR using the KOD-PLUS DNA polymerase (Toyobo, Osaka, Japan) [26]. Two 1-kb DNA fragments of the promoter and the coding sequence from the start codon of *wA* were amplified with LU/LL and RU/RL primer pairs from the RIB40 genomic DNA template. Similarly, a 1-kb DNA fragment of the *kojA* promoter was amplified with PU_PkojA/PL_PkojA primer pair from the RIB40 genomic DNA. A 4.7-kb DNA fragment of *AnniaD* including the promoter and the terminator was amplified as a selectable marker with PU_AnniaD/PL_AnniaD primer pair from *A. nidulans* A851 genomic DNA. These four DNA fragments were mixed and were subjected to fusion PCR with LU/RL primer pair (Additional file 1: Figure S2). The resultant fragment harboring the *wA* gene portion in which the promoter was replaced with the *kojA* promoter was introduced to the *ΔkojA_niaD-* strain by transformation. The *ΔkojA_PkojA::wA* stain was selected by picking transformants grown on CD agar medium and confirmed by both PCR with LU/RL primer pair and Southern hybridization.

The control strain, *ΔkojA*, was made by complementing the *niaD* gene obtained from *A. oryzae* with the *ΔkojA_niaD-* strain. A 5.4-kb DNA fragment of *niaD* including 0.9–1 kb regions of the promoter and the terminator was amplified with PU_niaD/PL_niaD primer pair from *A. oryzae* RIB40 genomic DNA and used for the transformation of *ΔkojA_niaD-* (Additional file 1: Figure S3)*.* The *ΔkojA* strain was selected by picking transformants grown on CD agar medium and confirmed by PCR with PU_niaD/PL_niaD primer pair.

### Southern hybridization

Aliquots (3 μg) of genomic DNA were digested with the restriction enzyme (*Hin*dIII or *Hinc*II), fractionated on a 0.7% agarose gel. Digoxigenin (DIG)-labeled probe (approximately 800 bp) was prepared using a PCR DIG Probe Synthesis Kit (Roche Applied Science, Mannheim, Germany) with the primers SBU and SBL. The probe preparation, hybridization, and signal detection were performed as described previously [3].

### Extraction of the yellow-colored compound, confirmation by UV-Vis spectrometry and UPLC-MS, and the quantification

To analyze the yellow-colored compound secreted from the *ΔkojA_PkojA::wA* strain, 30 mL of culture supernatant after liquid culture was extracted with 20 mL of ethyl acetate. The yellow-colored compound was extracted in the ethyl acetate fraction. The separated ethyl acetate fraction was concentrated in vacuo using a rotary evaporator, and the residue was dissolved in ethanol (1.5 mL). A small portion was added to 1 mL of Milli-Q water, and then UV-Vis spectrometry was performed by monitoring absorbance in the wavelength range from 200 nm to 500 nm using a UV-Vis spectrophotometer (Shimadzu UV-1800; Tokyo, Japan). Then, another small portion was re-dissolved with ethyl acetate and 1 μL of the solution was evaluated by ultraperformance liquid chromatography (UPLC)-mass spectrometry (MS) using the Waters Xevo TQD, Acquity UPLC H class equipped with an Waters Acquity UPLC BEH C18 (1.7 μm, 2.1 × 50 mm) column (Waters, Milford, MA, USA). 15% acetonitrile in Milli-Q water over 0–0.5 min and a subsequent linear gradient of 15 to 100% acetonitrile in Milli-Q water containing 0.1% formic acid over 0.5–15 min at a flow rate of 0.4 mL/min were used as the mobile phase. The column temperature was set to 40 °C. The yellow-colored compound was detected by monitoring absorbance at 280 nm. Because other compounds with absorbance at 280 nm, such as nucleic acids and proteins, were removed at the extraction step with ethyl acetate, the yellow-colored compound was detected with high sensitivity.

For the quantification of the yellow-colored compound, 500 μL of the culture supernatant was sampled and filtered using Ultrafree-MC-HV Centrifugal Filters Durapore-PVDF 0.45 μm (Millipore, Burlington, MA, USA). Then, absorbance at 406 nm was measured according to the second largest peak of UV spectrum at this wavelength. Since the culture supernatant was directly measured, this wavelength was used for precise quantification.

As a standard, the naphthopyrone compound YWA1, a product of polyketide synthase encoded by *wA* of *A. nidulans,* was similarly evaluated by both UV-Vis spectrometry and UPLC-MS [22].

### Quantitative reverse transcription polymerase chain reaction (qRT-PCR)

Primers named “real_fwd” and “real_rev” were used for the quantification of gene expression levels. Expression level of the histone gene (AO090012000496) was used for normalizing the expression level of each gene [27]. RNA extraction and the subsequent qRT-PCR were performed as described previously [3].

## Supplementary information

**Additional file 1: Figure S1.** Time-course shift in *enoA* expression in the wild-type RIB40 strain. Expression level at each time point relative to that on day 2 of RIB40 is shown. Error bars represent mean ± standard deviation (*n* = 3). The result of a significance test (Student’s t-test) between day 2 and the other date is shown above each bar. ** represent *p* < 0.01. **Figure S2.** Construction of the DNA fragment for replacement of *wA* promoter with *kojA* promoter in *A. oryzae*. The 7794 bp long DNA fragment was constructed for the replacement. Primers used for the construction and clone check are shown as arrows. **Figure S3.** Construction of the DNA fragment for complementing the *niaD* gene to the *A. oryzae ΔkojA_niaD-* where 769 bp long region was deleted at the original *niaD* locus. The 5398 bp long DNA fragment was constructed. Primers used for the construction and clone check are shown as arrows. **Figure S4.** UV-Vis spectra of the YWA1 standard (A) and yellow-colored compound produced by the *ΔkojA_PkojA::wA* strain (B). **Fig. S5.** Time-course shift of the *kojA* promoter activities. The *kojA* promoter activity evaluated as *kojA* expression level at the *kojA* locus in the wild-type RIB40 strain (orange) and the one evaluated as *wA* expression level at the *wA* locus in the *ΔkojA_PkojA::wA* strain (blue) are compared. Both expression levels are normalized by the expression level of the histone gene as an internal standard and then compensated expression level at each time point relative to that on day 2 of RIB40 is shown. Error bars represent standard deviation (n = 3). Result of a significance test (Student’s t-test) between day 2 of RIB40 and the other date is shown above each bar. ** represent *p* < 0.01. **Table S1.** DNA primers used in this study. Tails of primers are shown in lower case letters.

## Data Availability

The datasets used and/or analyzed during the current study are available from the corresponding author on reasonable request.

## Abbreviations

*A. nidulans*: *Aspergillus nidulans*
*A. niger*: *Aspergillus niger*
*A. oryzae*: *Aspergillus oryzae*
CD: Czapek–Dox
cDNA: complementary DNA
DIG: Digoxigenin
HPLC: High performance liquid chromatography
PCR: Polymerase chain reaction
PKS: Polyketide synthase
qRT-PCR: quantitative reverse transcription polymerase chain reaction
UPLC-MS: Ultraperformance liquid chromatography-mass spectrometry
UV-Vis: Ultraviolet–visible
