# Supplementary material for: Use of the kojA promoter, involved in kojic acid biosynthesis, for polyketide production in Aspergillus oryzae: implications for long-term production
Source: BMC Biotechnol. 2019 Oct 26;19:70. doi: 10.1186/s12896-019-0567-x (PMC6814975; doi:10.1186/s12896-019-0567-x)
Supplement: Supplementary file 1 — Additional file 1: Figure S1. Time-course shift in enoA expression in the wild-type RIB40 strain. Expression level at each time point relative to that on day 2 of RIB40 is shown. Error bars represent mean ± standard deviation (n = 3). The result of a significance test (Student’s t-test) between day 2 and the other date is shown above each bar. ** represent p < 0.01. Figure S2. Construction of the DNA fragment for replacement of wA promoter with kojA promoter in A. oryzae. The 7794 bp long DNA fragment was constructed for the replacement. Primers used for the construction and clone check are shown as arrows. Figure S3. Construction of the DNA fragment for complementing the niaD gene to the A. oryzae ΔkojA_niaD- where 769 bp long region was deleted at the original niaD locus. The 5398 bp long DNA fragment was constructed. Primers used for the construction and clone check are shown as arrows. Figure S4. UV-Vis spectra of the YWA1 standard (A) and yellow-colored compound produced by the ΔkojA_PkojA::wA strain (B). Fig. S5. Time-course shift of the kojA promoter activities. The kojA promoter activity evaluated as kojA expression level at the kojA locus in the wild-type RIB40 strain (orange) and the one evaluated as wA expression level at the wA locus in the ΔkojA_PkojA::wA strain (blue) are compared. Both expression levels are normalized by the expression level of the histone gene as an internal standard and then compensated expression level at each time point relative to that on day 2 of RIB40 is shown. Error bars represent standard deviation (n = 3). Result of a significance test (Student’s t-test) between day 2 of RIB40 and the other date is shown above each bar. ** represent p < 0.01. Table S1. DNA primers used in this study. Tails of primers are shown in lower case letters. [file 12896_2019_567_MOESM1_ESM.pdf]

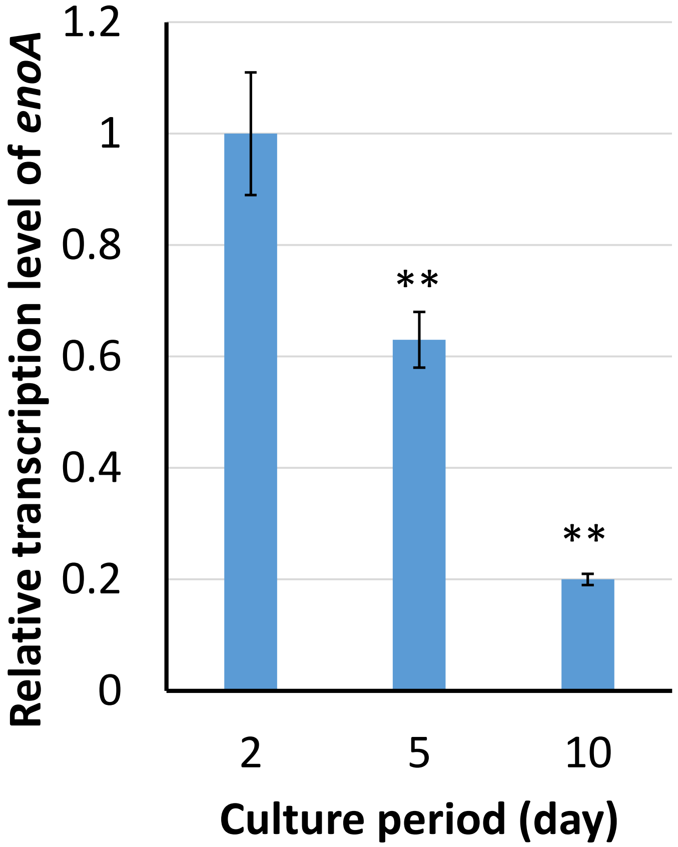

Supplementary Figure S1. Time-course shift in *enoA* expression in the wild-type RIB40 strain. Expression level at each time point relative to that on day 2 of RIB40 is shown. Error bars represent mean ± standard deviation (n = 3). The result of a significance test (Student's t-test) between day 2 and the other date is shown above each bar. ** represent $p < 0.01$.

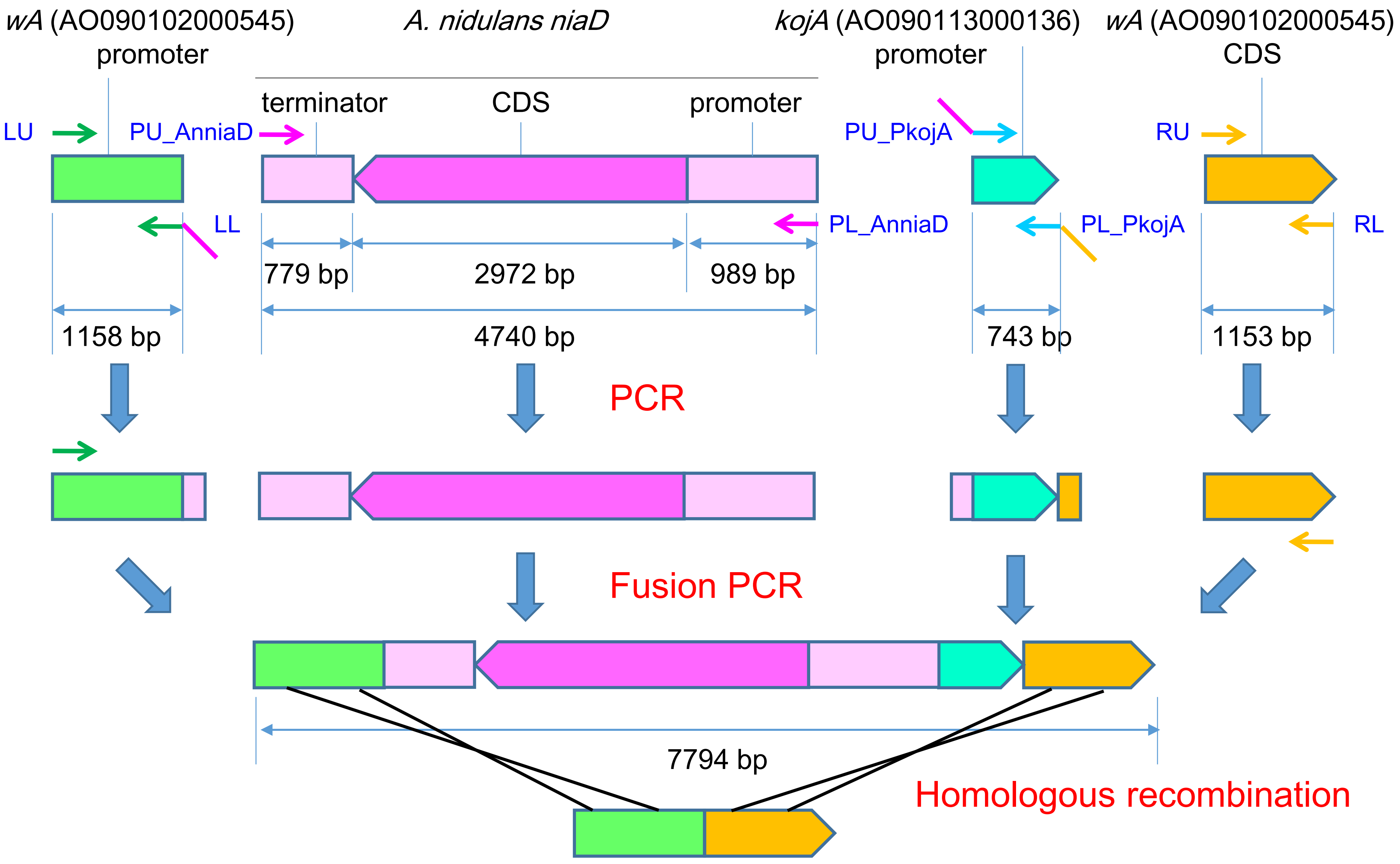

Supplementary Figure S2. Construction of the DNA fragment for replacement of *wA* promoter with *kojA* promoter in *A. oryzae*. The 7794 bp long DNA fragment was constructed for the replacement. Primers used for the construction and clone check are shown as arrows.

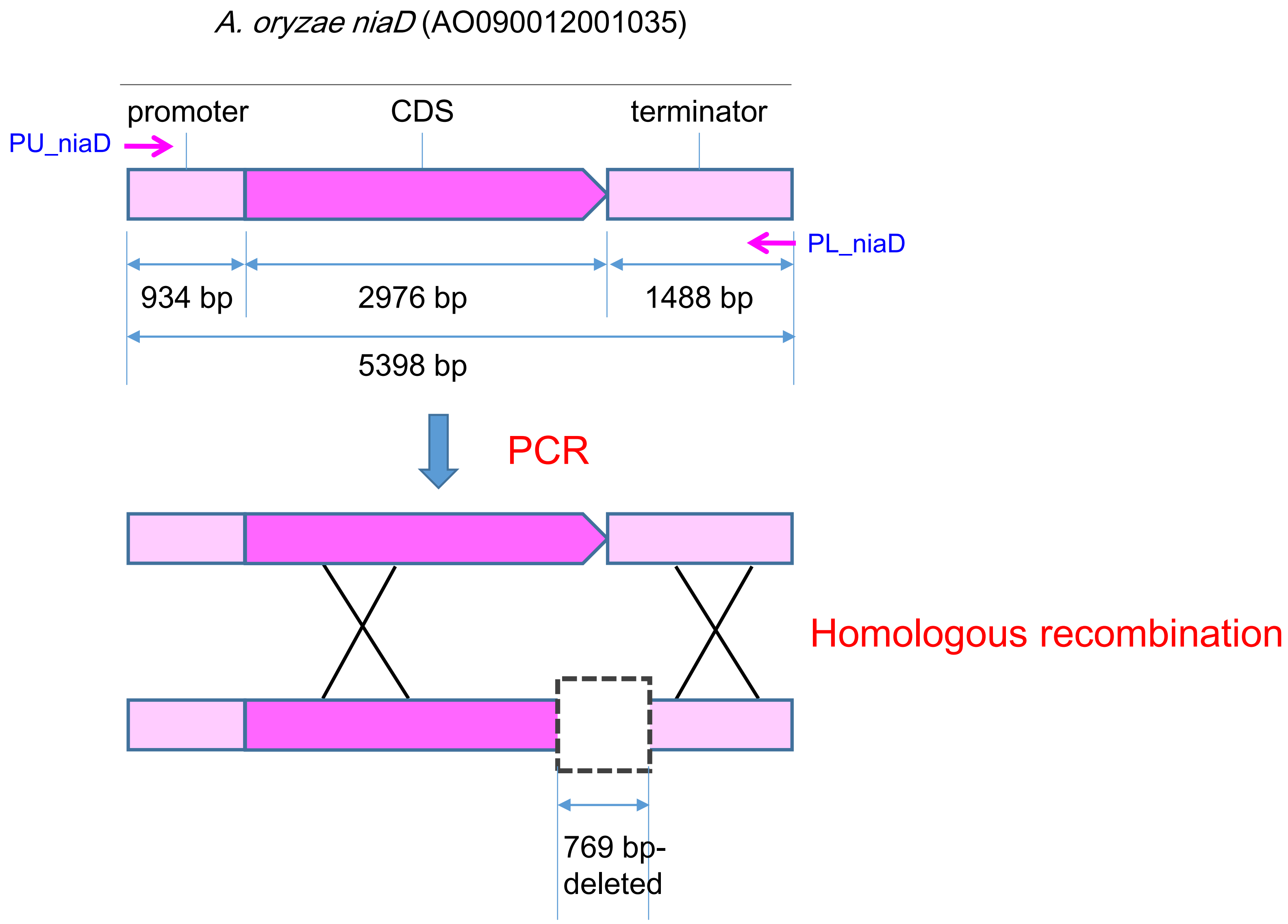

Supplementary Figure S3. Construction of the DNA fragment for complementing the *niaD* gene to the *A. oryzae ΔkojA_niaD-* where 769 bp long region was deleted at the original *niaD* locus. The 5398 bp long DNA fragment was constructed. Primers used for the construction and clone check are shown as arrows.

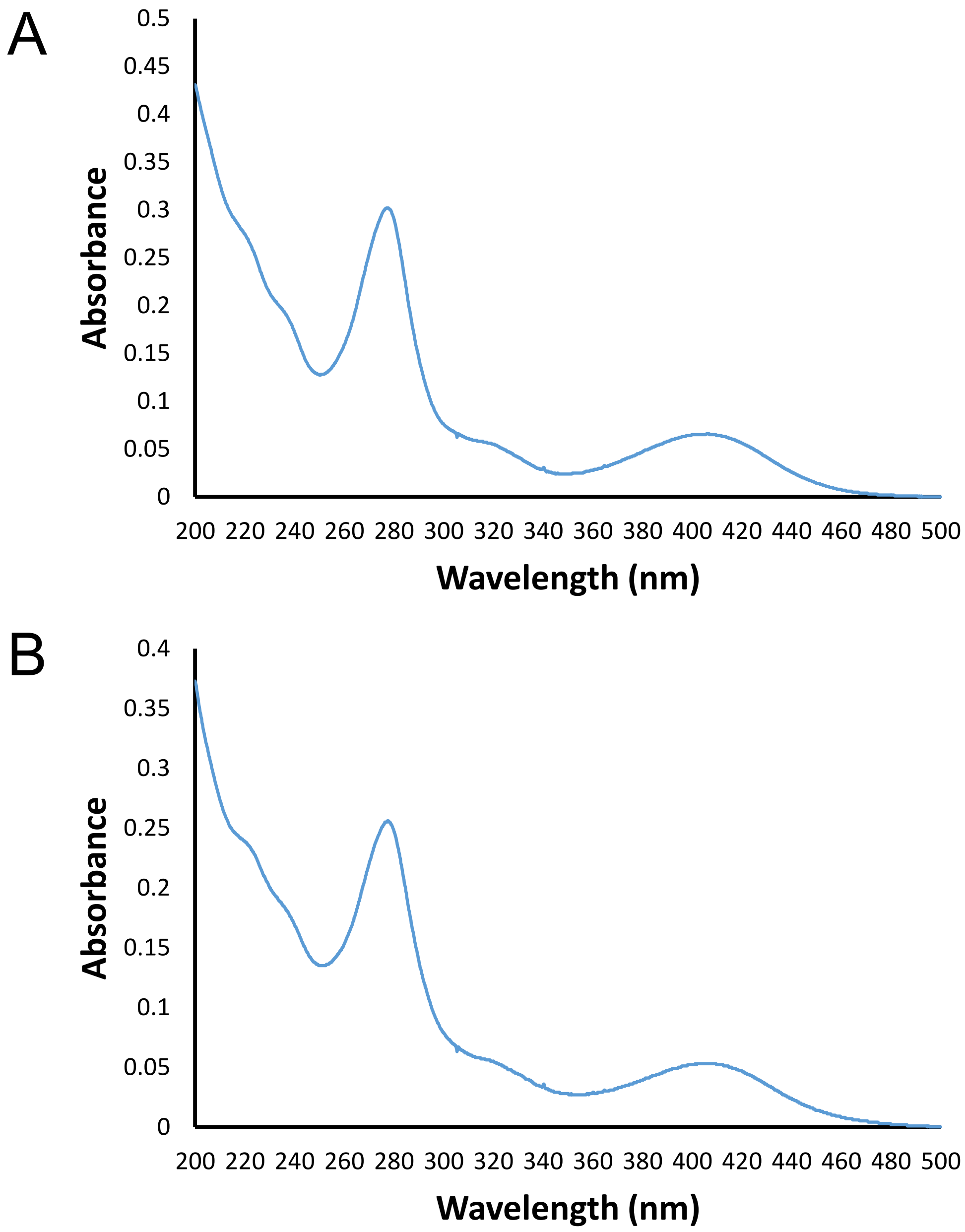

Supplementary Figure S4. UV-Vis spectra of the YWA1 standard **(A)** and yellow-colored compound produced by the *ΔkojA_PkojA::wA* strain **(B)**.

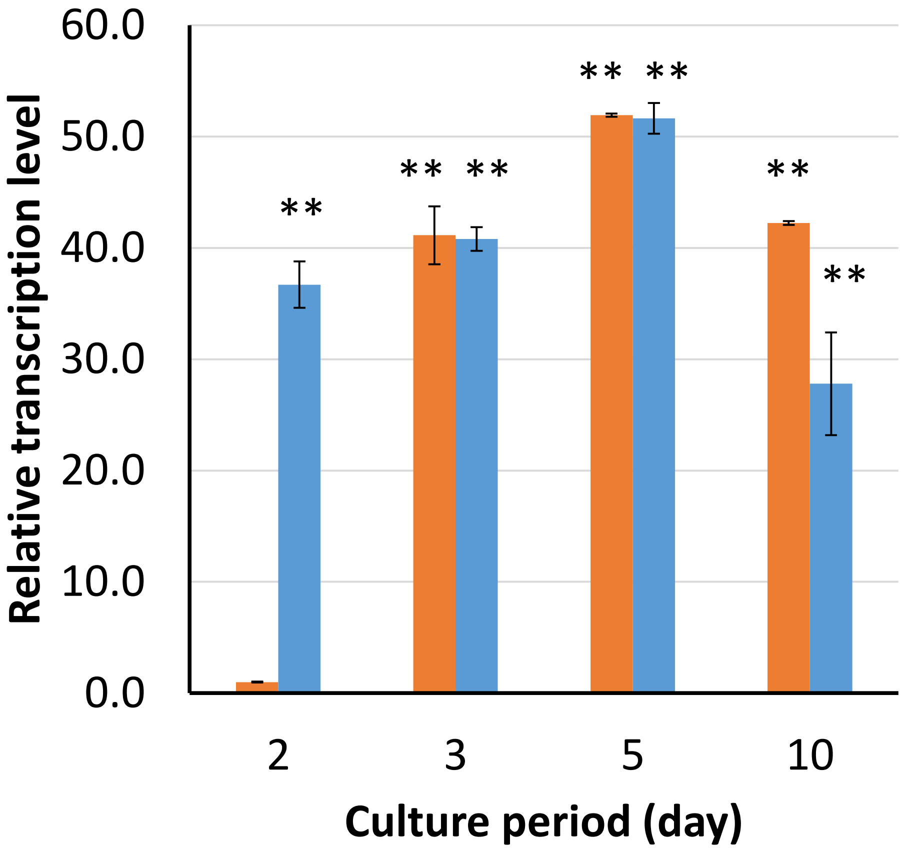

Supplementary Figure S5. Time-course shift of the *kojA* promoter activities. The *kojA* promoter activity evaluated as *kojA* expression level at the *kojA* locus in the wild-type RIB40 strain (orange) and the one evaluated as *wA* expression level at the *wA* locus in the *ΔkojA_PkojA::wA* strain (blue) are compared. Both expression levels are normalized by the expression level of the histone gene as an internal standard and then compensated expression level at each time point relative to that on day 2 of RIB40 is shown. Error bars represent standard deviation (n = 3). Result of a significance test (Student's t-test) between day 2 of RIB40 and the other date is shown above each bar. ** represent $p < 0.01$.

Supplementary Table S1. DNA primers used in this study

Name	Sequence (5' to 3')
LU	CTCCGATGTTACATGCAACCTGAT
LL	gtcagcggccgcatccctgcGTTGGGTCAAGAAAGTGCCAGTCA
PU_AnniaD	gcagggatgcggccgctgacTCGCCCTCAGCGACTACTTCTTCCA
PL_AnniaD	ccgctgctaggcgcgccgtgCTAGAGCCGCTTGACGATAATGAG
PU_PkojA	cacggcgcgcctagcagcggTACGCTGCACTACAGGGTTATCGC
PL_PkojA	gccgcgtggcccctccatTATGAAGAGGCAGGTAGTTATAGTCTAG
RU	taactacctgcctcttcataATGGAGGGGCCACGCGGCGTCTAT
RL	ATCCCGGCTTTCGTAAGAGCTGTA
PU_niaD	CCTCAGGTTTCACCACAGGTCATA
PL_niaD	GGGAACCACAAAGATCTTGAAGTA
SBU_PkojA	GTTCTTCCAGAGATGCTTTC
SBL_PkojA	ATTATGGCAGCTTTCGATGA
SBU_niaD	CAAGTGTTGCAGCAGTACGA
SBL_niaD	GGTGCGTACATTTGCCTTTG
real_fwd_wA	AGAAGCTGCCGCAGAGATTG
real_rev_wA	ATGCGTCGAGAGCGTCGATA
real_fwd_kojA	GCCAAGTTAAAGACCACATC
real_rev_kojA	CGCAATGTCATTGATCAGAG
real_fwd_histone	TCTTGCGTGACAACATCCA
real_rev_histone	AGATACGCTTGACACCACCA

Tails of primers are shown in lowercase letters.
